# Antagonistic Antibody Targeting TNFR2 Inhibits Regulatory T Cell Function to Promote Anti-Tumor Activity

**DOI:** 10.3389/fimmu.2022.835690

**Published:** 2022-02-16

**Authors:** Yonglin Chen, Manxue Jia, Sharon Wang, Sherry Xu, Nanhai He

**Affiliations:** Department of Biosciences, Adlai Nortye USA Inc., North Brunswick, NJ, United States

**Keywords:** TNFR2, antagonist, regulatory T cells, tumor microenvironment, cancer immunotherapy

## Abstract

Infiltration of regulatory T cells (Tregs) in the tumor microenvironment suppresses anti-tumor immune response, and promotes tumor progression. Tumor necrosis factor receptor‐2 (TNFR2), which is highly expressed on Tregs, activates Tregs through nuclear factor kappa B (NF-κB) pathway. Moreover, TNFR2^+^ Tregs have been shown to be most suppressive among all Tregs populations in tumor. Due to the unique expression pattern and function of TNFR2 on Tregs, a TNFR2 blocking antibody is expected to compromise Tregs function, relieve Tregs-mediated immunosuppression, and hence to enhance anti-tumor immune response. AN3025 is an antagonistic anti-human TNFR2 (hTNFR2) antibody that is currently under preclinical development. This study investigates the immunomodulatory and anti-tumor activity of AN3025. AN3025 was generated through rabbit immunization with extracellular domain of human TNFR2 and subsequent humanization by complementarity-determining regions (CDRs) grafting. AN3025 binds to the extracellular domain of both human and cynomolgus with sub-nanomolar affinity and specificity, but not mouse or rat TNFR2. AN3025 inhibited tumor necrosis factor alpha (TNFα) induced cell death of hTNFR2-overexpressing Jurkat cells by competing with TNFα for binding to hTNFR2. In the Tregs/T effector co-culture assay, AN3025 increased T effector proliferation and enhanced interferon gamma (IFNγ) production. As a monotherapy, AN3025 significantly inhibited MC38 tumor growth in TNFR2 humanized mouse model. Subsequent flow cytometry (FACS) and immunohistochemistry (IHC) analysis revealed that administration of AN3025 led to decreased Tregs population, increased CD4^+^ and CD8^+^ T cell numbers in the tumor. The anti-tumor activity of AN3025 was dependent on the existence of CD4^+^ and CD8^+^ T cells, as depletion of CD4^+^ and CD8^+^ T cells abolished the anti-tumor activity of AN3025. In addition, AN3025 in combination with anti-PD-1 antibody demonstrated stronger *in-vivo* anti-tumor activity. The potent anti-tumor efficacy of AN3025, either as a monotherapy or in combination with anti-PD-1 antibody, supports its further clinical development for the treatment of various human tumors.

## Introduction

Immune-checkpoint inhibitors (ICIs) are showing great promise in cancer therapy. Programmed cell death protein 1 (PD-1), programmed cell death 1 ligand 1 (PD-L1) and cytotoxic T lymphocyte associated protein 4 (CTLA-4) checkpoint inhibitors are important milestones in the drug development for cancer therapy (1). Even though ICIs have demonstrated successful elicitation of anti-tumor activity in multiple cancer types (2), the percentage of cancer patients who can respond to ICIs treatments is still limited (3). The combination of a checkpoint inhibitor with chemotherapy or with another checkpoint inhibitor could further improve the efficacy and response rates (4, 5). However, while ICI monotherapies trigger immune-related adverse events (irAE), combination treatments substantially increased irAE (6). Therefore, the discovery and development of new immunotherapies for cancer treatment is an ongoing need.

Regulatory T cells (Tregs) play an important role in maintenance of immune homeostasis through inhibiting the activation of CD4^+^ T cells and CD8^+^ T cells to avoid excessive and abnormal immune response (7, 8). The immunosuppressive function of Tregs benefits the control and prevention of autoimmune diseases caused by immune responses against autologous antigen (9). Multiple non-cell based and cell-based therapies have been developed to enhance Tregs mediated immunosuppression for treatment of autoimmune diseases (9). Conversely, Tregs accumulation occurred in tumor microenvironment (10, 11), hampered the anti-tumor immune response and led to progression of cancer (12, 13). Furthermore, depletion of Tregs in murine tumor models induced anti-tumor response and inhibited tumor growth (14, 15). Based on the role of Tregs in cancer and the *in-vivo* studies, there has been great interest of developing immunotherapies that can block or deplete Tregs to enhance anti-tumor immunity (16, 17).

The tumor necrosis factor alpha (TNFα) is a pleiotropic cytokine exerting its function through TNF receptor 1 (TNFR1) or TNFR2. Unlike the extensive expression of TNFR1, expression of TNFR2 is mostly limited to immune cells (18). It is noteworthy that TNFR2 is highly expressed on Tregs compared with conventional CD4^+^ or CD8^+^ T cells (19). Moreover, TNFR2^+^ Tregs has been demonstrated to be the most suppressive Tregs subpopulation (20). The TNFR2 expression by Tregs was largely shown to exert protective functions in autoimmunity (21) due to the fact that TNFR2 activation by TNFα (22) or TNFR2 agonists (23, 24) results in enhanced Tregs expansion and suppressor function. Therefore, expansion of Tregs *via* TNFR2 activation (i.e., agonist antibody) has been a promising therapeutic strategy for autoimmune diseases (21). In contrast, TNFR2 antagonists inhibited the *in-vitro* proliferation of Tregs derived from ovarian cancer patients and Sézary syndrome patients while enabling the T effector cells expansion (25, 26). The role of TNFR2 in cancer was also studied with murine tumor models. In TNFR2 deficiency mice, liver metastases of colon (MC-38) and lung (H-59) carcinoma cells decreased significantly compared in wild-type mice (27). Further, the reduction in liver metastases in TNFR2 deficiency mice could be abolished by reconstitution with normal bone marrow, suggesting the TNFR2 expression on immune cells impaired the anti-tumor activity (27). Similar reduction in lung metastases of B16F10 melanoma cells was observed in bone marrow chimeric mice which specifically lacked TNFR2 receptor on immune cells (28). In these studies, reduced tumor metastases were accompanied with decreased Tregs in the metastatic sites (27, 28). These observations indicated TNFα-TNFR2 interaction is essential for the immunosuppression mediated by Tregs in cancer.

Overall, TNFR2 is emerging as an attractive target for cancer treatment. TNFR2 antagonistic antibody appears to be a promising approach to inhibit Tregs function in tumor patients and to induce anti-tumor immune response (2, 29). In this study, we characterized AN3025, a humanized antibody against human TNFR2 (hTNFR2), in aspect of binding affinity, species cross reactivity and capacity to block TNFR2 activation. Further, *in-vivo* anti-tumor activity of AN3025 as monotherapy or in combination with anti-PD-1 antibody were evaluated.

## Materials and Methods

### AN3025 Generation

AN3025 was generated through rabbit immunization with extracellular domain (ECD) of human TNFR2 and subsequent humanization by complementarity-determining regions (CDRs) grafting. Briefly, New Zealand white rabbits (specific pathogen-free) were immunized with extracellular domain of human TNFR2 (Acro Biosystem, TN2-H5227). Single-chain variable fragment phage library was constructed with rabbit VH and VL regions amplified from rabbit spleen cDNA. Monoclonal phage enzyme-linked immunosorbent assay (ELISA) was performed to identify binders against human TNFR2. Chimeric anti-human TNFR2 antibody was generated through fusion of selected rabbit VH to a human IgG_1_ constant region and fusion of selected rabbit VL to a human IgG_1_ kappa constant domain. The chimeric antibody was further humanized by inserting the rabbit CDRs into human germline frameworks.

### Animals and Treatment

Six- to ten-week-old humanized female B-hTNFR2 mice (Biocytogen) were housed in the specific-pathogen-free (SPF) barrier facility of the Animal Center of Beijing Biocytogen Co., Ltd in individual ventilated cage. The experimental animals were acclimatized for 7 days before being used in experiments. All experimental animal procedures were in accordance to the Institutional Animal Care and Use Committees (IACUC) guidance. Mice were euthanized with CO_2_. All efforts were made to minimize animals’ distress and pain. Murine colon cancer MC38 cells were subcutaneously implanted into homozygous B-hTNFR2 mice. Mice were grouped when tumor volume reached approximately 100 mm^3^. Then mice were treated with PBS, anti-mouse PD-1 antibody (BioXcell, BP0146), AN3025 or combination of anti-mouse PD-1 antibody and AN3025 through i. p. injection. Anti-mouse CD4 (BioXcell, BP0003-1) and anti-mouse CD8 (BioXcell, BP0004-1) were used at 0.3mg per mouse per i.p. injection for depletion of CD4^+^T cells and CD8^+^ T cells. Animal well-being and behaviors were monitored during the experiment process. Animal body weight and tumor volume were measured twice a week. The anti-tumor efficacy is expressed as tumor growth inhibition in terms of tumor volume (TGI_TV_). The TGI_TV_ in percent was calculated as below:


$${\text{TGI}}_{\text{TV}}(\%)=[1-({\text{T}}_{\text{i}}-{\text{T}}_{0})/({\text{C}}_{\text{i}}-{\text{C}}_{0})]\times 100\%;$$

Where T_i_= mean tumor volume of drug-treated group on the final day of the study, T_0_= mean tumor volume of drug-treated group on first dosing day, C_i_= mean tumor volume of control group on the final day of the study, C_0_= mean tumor volume of control group on the first dosing day.

### ELISA

Human TNFR2 (Acro Biosystem, TN2-H5227), human TNFR1 (Acro Biosystem, TN1-H5222), cynomolgus TNFR2 (Sino Biological,90102-C08H), mouse TNFR2 (R&D systems, 426-R2-050/CF) or rat TNFR2 (R&D systems, 8348-R2-050) was coated on high binding polystyrene flat bottom micro-titer plates (Thermo Scientific, 3455). Plates were incubated at 4°C overnight. Plates were washed with PBST (wash buffer 0.1%) and blocked with 1% BSA (Fisher Scientific, BP1600-100) in PBS for 1 hour at 37°C. AN3025 or human IgG_1_, κ (BioxCell, BE0297) was titrated, distributed in the wells and incubated at 37°C for 1 hour. Plates were washed with PBST (wash buffer 0.1%) and incubated with goat anti-human IgG Fc secondary antibody-horseradish peroxidase (HRP) conjugate (Invitrogen, A18817) for 1 hour at 37°C. Plates were washed and developed using TMB substrate solution (eBioscience,00-4201-56) and stopped with ELISA stop solution (Invitrogen, SS04). The level of bound antibodies was determined by reading absorbance at 450 nm. Mouse anti-human TNFR1 antibody (ThermoFisher Scientific, MA1-81005) and goat anti mouse IgG secondary antibody-HRP conjugate (Invitrogen, 62-5520) were used as positive control for human TNFR1 binding. Armenian hamster anti-mouse TNFR2 antibody (InVivoMab, BE0247) and HRP-goat anti-armenian hamster IgG (H+L) secondary antibody (Invitrogen, PA1-32045) were used as positive control for mouse TNFR2 binding. TNFR2 polyclonal antibody (Invitrogen, PA5-80159) and HRP conjugated Goat anti-Rabbit IgG Fc Secondary Antibody (Invitrogen, A16116) were used as positive control for rat TNFR2 binding.

### Competitive ELISA

Human TNFα (Gibco, PHC3011) was biotinylated using an EZ-Link™ Micro Sulfo-NHS-Biotinylation Kit (ThermoFisher Scientific, 21925) according to manufacturer’s instructions. Human TNFR2 (Acro Biosystem, TN2-H5227) was coated on high binding polystyrene flat bottom micro-titer plates (Thermo Scientific, 3455). Plates were incubated at 4°C overnight. Plates were washed with PBST (wash buffer 0.1%) and blocked with 1% BSA (Fisher Scientific, BP1600-100) in PBS for 1 hour at 37°C. AN3025 or human IgG_1_, κ (BioxCell, BE0297) was titrated, distributed 50μL per well and incubated for 1 hour at 37°C. 50μL biotinylated human TNFα was added per well to achieve the final TNFα concentration at 100ng/ml. After another 1-hour incubation, plates were washed with PBST (wash buffer 0.1%). Streptavidin-HRP conjugate (Thermo Scientific, N504) was distributed to detect bound biotinylated human TNFα. After 1-hour incubation of secondary antibody at 37°C, plates were washed with PBST (wash buffer 0.1%). Plates were developed using TMB substrate solution (eBioscience,00-4201-56) and stopped with ELISA stop solution (Invitrogen, SS04). The level of bound biotinylated human TNFα was determined by reading absorbance at 450 nm.

### Stable Single Cell Clone Establishment

Full length human TNFR2 was cloned into pLVX-EF1a-IRES-Puro vector (Takarabio, 631253). Lentivirus production and target cell transduction were performed according to manufacturer’s instructions. Briefly, human TNFR2-lentivector was mixed with ViraPower™ Packaging Mix (Invitrogen, K4975-00) and transfected 293T cell line (Sigma-Aldrich, 12022001) using Lipofectamine 2000 Reagent (Invitrogen, 11668-019) to produce a lentiviral stock. Jurkat E6-1 cells (ATCC, TIB-152) were transduced with human TNFR2-lentivirus in the presence of 8µg/mL polybrene (EMD, Millipore, TR-1003-G) and selected with 1µg/mL puromycin (Gibco, A11138-03). The pool of surviving cells was expanded. Single clones were generated from the hTNFR2 expressing pooled cells by single cell plate sorting on cell sorter (Sony, SH800). The parental Jurkat cells and single cell clones were blocked with human TruStain FcX™ (Biolegend, 422302) for 10 minutes at room temperature according to manufacturer’s instructions. Then cells were stained with phycoerythrin (PE) conjugate anti-human TNFR2 antibody (ThermoFisher Scientific, TNFR7504) for 30 minutes at 4°C. Cells were analyzed by flowcytometry (Sony, SA3800). Data was analyzed with Flowjo software.

### Cell Viability Assay

Parental Jurkat E6-1 cells or TNFR2 overexpressing Jurkat single clone cells were plated in opaque 96 well plates with 10^4^ cells in 100μL complete RPMI medium per well. Gradient concentration of human TNFα (Gibco, PHC3011) was added. The cell viability after 24 hours culture was measured with Promega CellTiter-Glo^®^ Luminescent Cell Viability Assay (Promega, G7570) according to manufacturer’s instructions. AN3025 or human IgG_1_, κ (BioxCell, BE0297) was added to the hTNFR2 overexpressing Jurkat cells by dose titration along with constant human TNFα (0.5 ng/ml). The cell viability after 24 hours culture was measured with Promega CellTiter-Glo^®^ Luminescent Cell Viability Assay (Promega, G7570).

### Tregs-T Effector Cell Co-culture Assay

Human peripheral blood mononuclear cells (PBMC) were isolated from normal healthy donor blood (New York Blood Center) with Ficoll (GE HealthCare, 17-1440-03) according to manufacturer’s instructions. Human CD4^+^ T cells were isolated from PBMC with human CD4^+^ T cell isolation kit (StemCell Technologies, 17952). Tregs were induced with human Tregs differentiation kit (R&D Systems, CDK006) according to manufacturer’s instructions. Autologous CD4^+^ T cells were label with carboxyfluorescein succinimidyl ester (CFSE, Invitrogen, C34554). Tregs and autologous CD4^+^T cells were co-cultured with human T-activator CD3/CD28 Dynabeads (Gibco,11131D) for 4 days in the presence of AN3025 or human IgG_1_, κ (BioxCell, BE0297). Proliferation of CD4^+^ T effector cells was evaluated with CFSE^lo^ population by flowcytometry (Sony, SA3800). Interferon gamma (IFNγ) production in the supernatant was detected by ELISA kit (R&D Systems, DY285B).

### Tumor Infiltrating Lymphocytes (TILs) Analysis

MC38 tumor tissues were collected at the end of MC38 tumor model study and minced into pieces. Single cell suspension was obtained by digesting minced tumor tissues with tumor dissociation kit (Miltenyi Biotec,130-096-730) in GentleMACS Octo dissociator. Cells were blocked with purified anti-mouse CD16/32 (Biolegend,101302) and stained with fluorescent antibodies against CD45 (Biolegend, 103116), CD3 (Biolegend, 100328), CD4 (Biolegend, 116004), Foxp3 (eBioscience, 25-5773-82) and fixable viability dye eFluor ™506 (eBioscience, 65-0866-14). Intracellular staining was performed with Foxp3/transcription factor staining buffer set (eBioscience, 00-5523-00) according to manufacturer’s instructions. The tumor infiltrating lymphocytes were analyzed with flowcytometry.

To perform the TILs analysis by immunohistochemistry (IHC), MC38 tumors were collected from B-hTNFR2 mice and fixed with formalin and embedded in paraffin. Tissues sections (3-4μm thick) were taken on 3-aminopropyltriethoxysilane (APTS) coated glass slides then de-paraffinized in xylene followed by hydration in graded ethanol. Specimens were heated at 100°C for 20 min in 0.01M citrate buffer (pH 6.0) using an EZ antigen retriever system (Biogenex, USA) to retrieve antigen. Endogenous peroxidase was blocked by treatment with 0.3% hydrogen peroxide. Non--specific binding sites were blocked with a protein block. Sections were covered with primary antibodies against mouse CD4 (Cell signaling, 25229S, Rabbit IgG1) or CD8 (Cell signaling, 98941, Rabbit IgG1) and incubated in moist chamber overnight at 4°C. After labeling with primary antibody, slides were washed with TBS (pH 7.4), blocked with post primary block at room temperature. Sections were then washed twice in TBS followed by incubation with Novolink polymer at room temperature. After washing with TBS for three times, sections were treated with DAB chromogen (3, 3’-diaminobenzidine tetrahydrochloride) in the dark. Sections were counterstained with hematoxylin, dehydrated with ethanol and xylene, and mounted permanently with Di-n-butylPhthalate in Xylene (DPX). Negative control slides omitting the primary antibody were included in all batches. Scoring of immune stained positive TILs was done independently by at least two pathologists. CD4^+^ and CD8^+^ TILs were counted in five randomly selected fields at 40X magnification and the counts were averaged.

### Statistical Analysis

All data are shown as Mean ± SEM. Experimental data were analyzed with the student’s t-test to calculate the P values. Statistical analysis of the tumor volumes on the final day of experiments was performed *via* t-test. Statistical analysis of tumor growth was also performed *via* two-way ANOVA and the corresponding P values were listed in **Supplementary Table 1**. Values of P < 0.05 were considered statistically significant. *P<0.05; ** P<0.01; ***P<0.001.

## Results

### AN3025 Binds to Human and Cynomolgus TNFR2 Selectively

**Figure 1:**
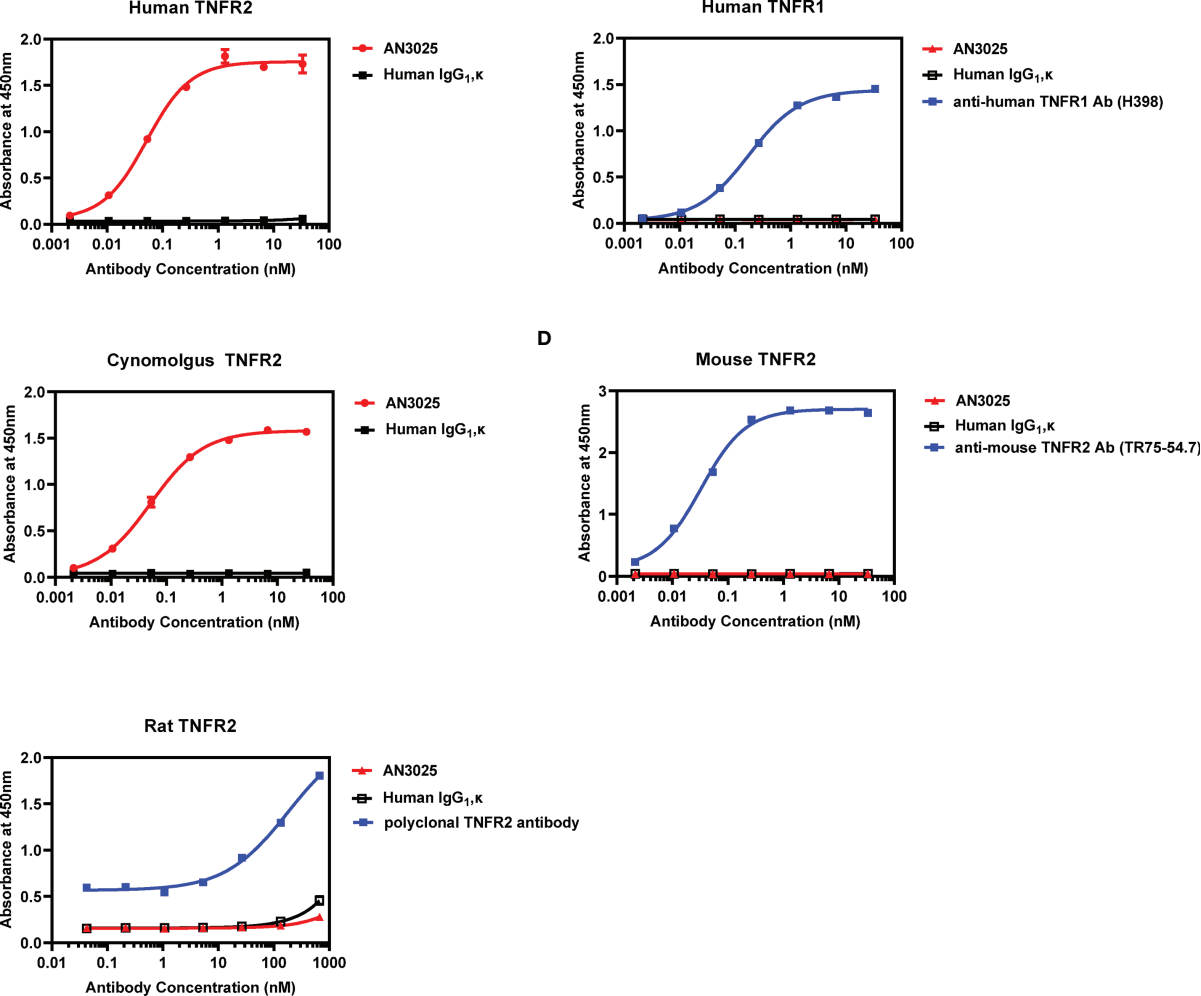
AN3025 binds to human TNFR2 and cynomolgus TNFR2 selectively. **(A)** Binding assay of AN3025 to human TNFR2 on plate by ELISA (EC_50_ = 0.052nM). **(B)** Binding assay of AN3025 to human TNFR1 on plate by ELISA. **(C)** Binding assay of AN3025 to cynomolgus TNFR2 on plate by ELISA (EC_50_ = 0.053nM). **(D)** Binding assay of AN3025 to mouse TNFR2 on plate by ELISA. **(E)** Binding assay of AN3025 to rat TNFR2 on plate by ELISA. The ELISA assays were tested in duplicates. Values were expressed as Mean ± SEM.

AN3025 was generated through rabbit immunization with extracellular domain of human TNFR2 and subsequent humanization by CDRs grafting. ELISA assay was performed to confirm and characterize its binding affinity and selectivity. AN3025 binds to human TNFR2 on plate with sub nanomolar affinity (EC_50_ = 0.052nM, **Figure 1A**) but not to human TNFR1 (**Figure 1B**). Species cross reactivity was also evaluated. AN3025 exhibited sub nanomolar affinity to cynomolgus TNFR2 (**Figure 1C**, EC_50_ = 0.053nM), but didn’t recognize mouse (**Figure 1D**) or rat TNFR2 (**Figure 1E**).

### AN3025 Inhibits TNFα-TNFR2 Mediated Jurkat Cell Death

**Figure 2:**
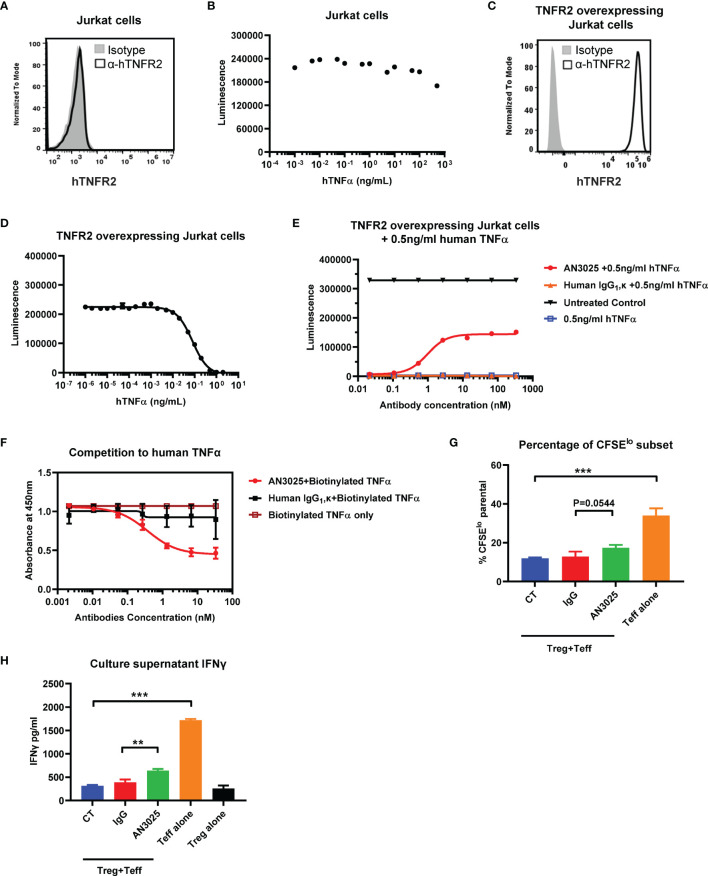
AN3025 inhibits TNFα-TNFR2 mediated cell death of hTNFR2 overexpressing Jurkat cell and enhances T effector cell function in Tregs/Teff co-culture assay. **(A)** Expression of human TNFR2 on wildtype Jurkat cells. **(B)** Viability of wildtype Jurkat cells after stimulation with titrated human TNFα. **(C)** Expression of human TNFR2 on established hTNFR2 overexpressing Jurkat single cell clone. **(D)** Viability of hTNFR2 overexpressing Jurkat cells after stimulation with titrated human TNFα. **(E)** Viability of hTNFR2 overexpressing Jurkat cells after stimulation with constant 0.5ng/mL human TNFα in the presence of titrated AN3025 or titrated human IgG_1_, κ. **(F)** Competitive ELISA binding assay of AN3025 with biotinylated human TNFα to human TNFR2 on plate. **(G, H)** Human iTregs were co-cultured with CFSE-labeled autologous CD4^+^ Teff cells for 4 days. **(G)** CFSE^lo^ T effector cell percentage was quantified by flow cytometry. **(H)** IFNγ in the supernatants was detected by ELISA assay. Values were expressed as Mean ± SEM. **P < 0.01; ***P < 0.001.

Human TNFα-TNFR2 signaling axis has been reported to induce cell death of TNFR2 overexpressing Jurkat cells but didn’t affect the viability of wildtype Jurkat cells lacking TNFR2 expression (30, 31). We firstly detected the expression of human TNFR2 on wildtype Jurkat cells and stimulated Jurkat cells with titrated human TNFα up to 1000ng/ml. Consistent with previous reports (30, 31), Jurkat cells didn’t express human TNFR2 (**Figure 2A**). Meanwhile, treatment with human TNFα didn’t show much effect on the Jurkat cell viability (**Figure 2B**). A hTNFR2 overexpressing Jurkat single cell clone was established by transducing Jurkat cells with hTNFR2-lentivirus. The overexpression of hTNFR2 on the single cell clone was confirmed with flowcytometry (**Figure 2C**). TNFR2 overexpressing Jurkat cells are highly sensitive to TNFα-induced cell death with 99% cell killing achieved at TNFα concentration as low as 2 ng/ml (**Figure 2D**).

Taken together, these results demonstrated the activation of TNFα-TNFR2 signaling pathway induced cell death of TNFR2 overexpressing Jurkat cells. This assay could be used to evaluate the antagonistic activity of AN3025 against the TNFα-TNFR2 signaling pathway. TNFR2 overexpressing Jurkat cells were treated simultaneously with constant concentration of 0.5ng/ml human TNFα and titrated human IgG_1_, κ control or AN3025. AN3025 rescued TNFR2 overexpressing Jurkat cells from TNFα-induced cell death through a dose-dependent way (**Figure 2E**), indicating AN3025 was capable to block the TNFα-TNFR2 signaling pathways.

### AN3025 Competes With Human TNFα for Binding of hTNFR2

To further investigate the mechanism by which AN3025 blocked the TNFα-TNFR2 signaling pathway, we next examined if AN3025 compete with human TNFα for receptor binding of hTNFR2. Human TNFR2 was coated on plate and incubated with human IgG_1_, κ control or AN3025. Biotinylated human TNFα was added. Bound human TNFα was detected with HRP-conjugated streptavidin. Pre-incubation with AN3025 decreased the TNFα binding to hTNFR2 compared with the IgG_1_, κ control (**Figure 2F**). These data suggest AN3025 blocked the TNFα-hTNFR2 signaling by competing with human TNFα for binding of TNFR2.

### AN3025 Enhances T Effector Cell Proliferation and IFNγ Production in Tregs/Teff Co-culture Assay

During the chronic development of cancer, induced Tregs (iTregs) arise from naive CD4^+^ cells in the periphery, infiltrate into the tumor microenvironment and suppress the effector T cells in aspects of cell proliferation and cytokines production (12). In addition, TNFR2 was reportedly to be highly expressed on Tregs (19), therefore we sought to evaluate whether AN3025 could affect the function of iTregs. To this end, iTregs were produced and subsequently co-cultured with CFSE stained autologous CD4^+^ T effector cells in the presence of AN3025 or human IgG_1_, κ control. AN3025 addition increased CD4^+^ T effector proliferation (**Figure 2G**, P=0.0544) and significantly elevated IFNγ production in the supernatant (**Figure 2H**). Thus, AN3025 is capable to inhibit iTregs mediated immune suppression, leading to enhanced immune activation of T effector cells.

### AN3025 Inhibits MC38 Tumor Growth in hTNFR2 Mouse Model as a Monotherapy

ELISA assay has demonstrated AN3025 lacks cross reactivity to mouse TNFR2 (**Figure 1D**). To determine the *in-vivo* anti-tumor activity of AN3025, TNFR2 humanized mice (B-hTNFR2) from Biocytogen were used for murine tumor model study. Leukocyte subpopulations, including T/NK cells, Tregs, B cells, DC, granulocytes, and monocytes/macrophages, were comparable in B-hTNFR2 mice to those in wild-type mice both in spleen and in lymph node (32). In addition, human TNFR2 instead of murine TNFR2 expression was confirmed on the spleen Tregs in B-hTNFR2 mice (32). Interestingly, one previous study (33) implied that human TNFα couldn’t function through mouse TNFR2, we therefore sought to test whether mouse TNFα could instead interact with human TNFR2, given the hTNFR2 humanized mice used in the *in-vivo* studies. Our data showed mouse TNFα induced a dose-dependent cell death of human TNFR2 overexpressing Jurkat cells to a similar extend as for human TNFα, indicating mouse TNFα can function through human TNFR2 (**Supplementary Figure 1**).

**Figure 3:**
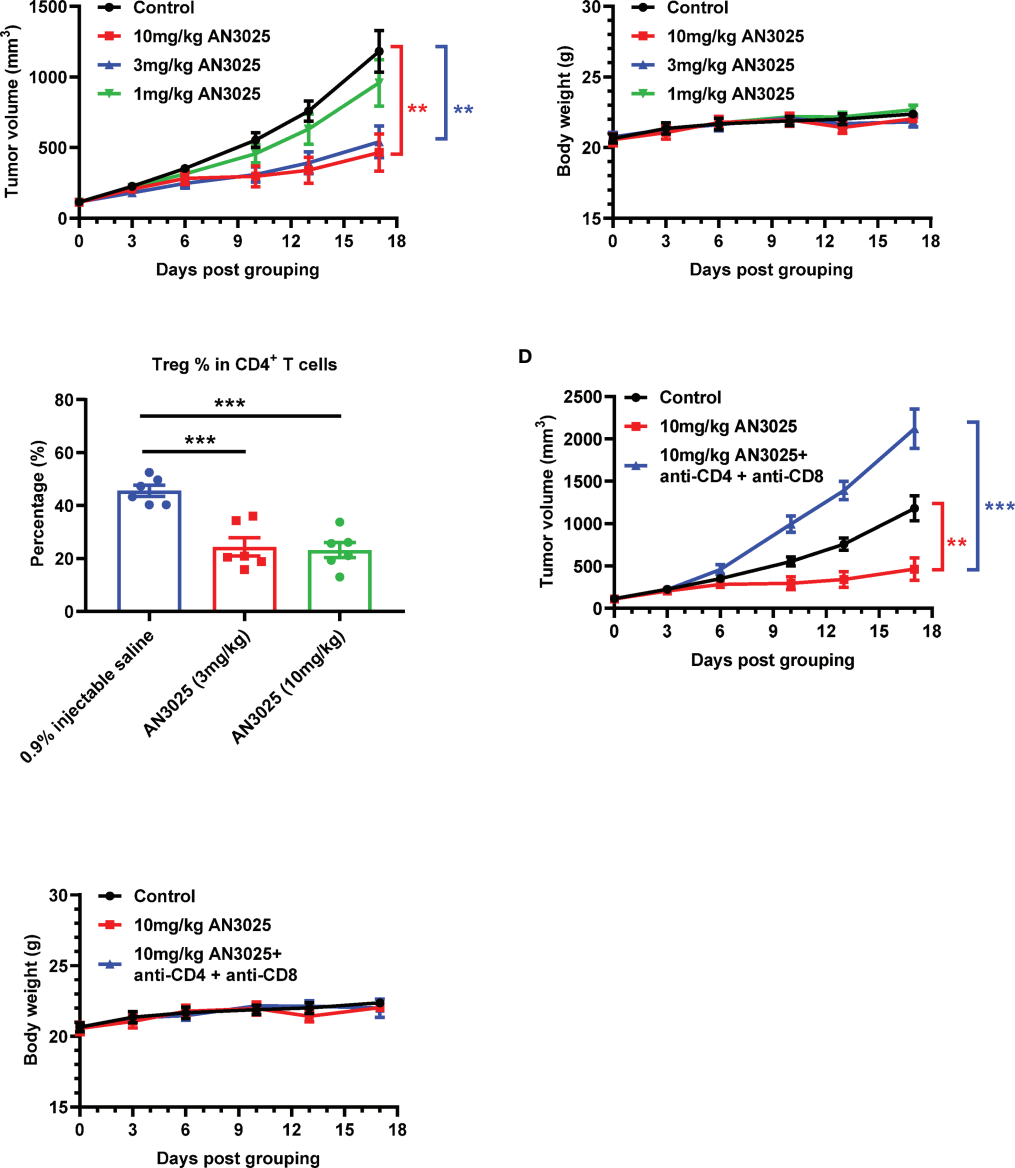
AN3025 significantly inhibits MC38 tumor growth as a monotherapy in hTNFR2 mouse model. **(A–E)** Murine colon cancer MC38 cells (5E5) were implanted subcutaneously into homozygous TNFR2 humanized mice (female). Mice were grouped when tumor volume reached approximately 100 mm^3^. **(A, B)** MC38 tumor bearing TNFR2 humanized mice were treated with AN3025 at the dosage of 10mg/kg, 3mg/kg or 1mg/kg every 4 days intraperitoneally for 5 doses in total. **(A)** Tumor volume measurement during the treatment (n=8 each group). **(B)** Body weight record during the treatment (n=8 each group). **(C)** MC38 tumor bearing TNFR2 humanized mice were treated with AN3025 at the dosage of 10mg/kg, 3mg/kg every 3 days intraperitoneally for 3 doses in total. Tregs (CD45^+^CD3^+^CD4^+^ Foxp3^+^) frequency in total CD4^+^ T cells in the MC38 tumors was quantified by flow cytometry (n=6 each group). **(D, E)** MC38 tumor bearing TNFR2 humanized mice were treated intraperitoneally with 10mg/kg AN3025 alone or combination of 10mg/kg AN3025, 0.3mg/mouse anti-mouse CD4 depletion antibody and 0.3mg/mouse anti-mouse CD8 depletion antibody every 4 days for 5 doses in total. **(D)** Tumor volume measurement during the treatment (n=8 each group). **(E)** Body weight record during the treatment (n=8 each group). Values were expressed as Mean ± SEM. **P < 0.01; ***P < 0.001.

MC38 (5E5) murine colon cancer cells were inoculated into B-hTNFR2 mice, and mice bearing MC38 tumor were later treated with AN3025 antibodies separately at 1 mg/kg, 3mg/kg or 10mg/kg. AN3025 treatments at dosage of 10mg/kg and 3mg/kg achieved significant tumor growth inhibition (TGI) at 67.2% and 60.0% respectively (**Figure 3A**). Meanwhile, treatment at 1mg/kg didn’t show tumor growth inhibition effect (**Figure 3A**). None of the three dosages induced significant body weight change in the experimental animals compared with the control group (**Figure 3B**).

To further investigate the anti-tumor mechanism of AN3025, we analyzed the Tregs frequency in the MC38 tumors after AN3025 treatments. Both treatments at 10mg/kg and 3mg/kg significantly decreased Tregs frequency in the tumors (**Figure 3C** and **Supplementary Figure 2**). This data demonstrated that AN3025 might inhibit either the proliferation or infiltration of Tregs in the tumor microenvironment, thus consequently activating the anti-tumor T cell response to exert anti-tumor activity. Consistently, RNA seq analysis (**Supplementary Figure 3**) of MC38 tumor tissues showed AN3025 treatment elevated expression of immune activation genes such as *ifn* (gene for IFNγ) and *gzmk* (gene for Granzyme K). In order to further test the immune dependency of AN3025 in controlling tumors, antibodies were used to deplete CD4^+^ T and CD8^+^ T cells simultaneously with AN3025 treatment. The anti-tumor activity of AN3025 was abolished by CD4^+^ and CD8^+^ T cells depletion (**Figure 3D**). No body weight change was observed with the indicated treatments (**Figure 3E**). Therefore, the anti-tumor activity of AN3025 is dependent on the existence of CD4^+^ and CD8^+^ effector T cells.

Besides the murine MC38 tumors, the anti-tumor activity of AN3025 as a monotherapy was also tested in melanoma B16F10 tumors with TNFR2 humanized mice. AN3025 (10mg/kg) achieved tumor growth inhibition at 46.6% in B16F10 tumor model without body weigh change in TNFR2 humanized mice (**Supplementary Figure 4**).

### AN3025 Enhances Anti-tumor Efficacy of Anti-mouse PD-1 Antibody in a Combination Study

**Figure 4:**
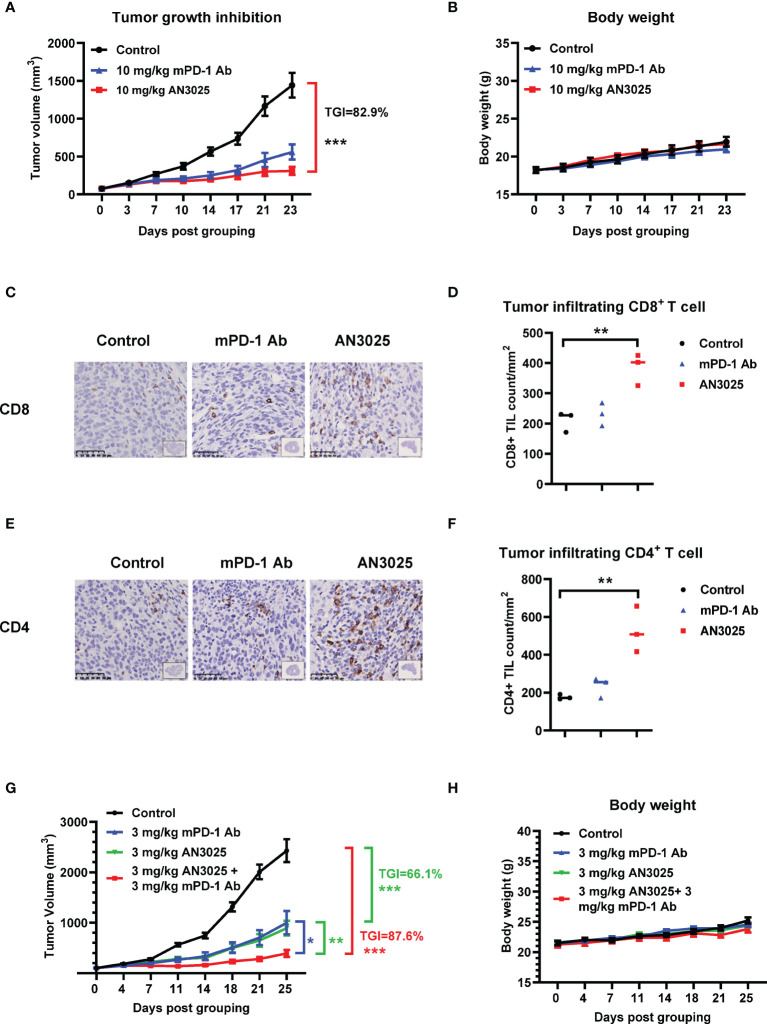
AN3025 enhances anti-tumor efficacy of mouse PD-1 antibody in combination study. **(A–H)** Murine colon cancer MC38 cells (5E5) were implanted subcutaneously into homozygous TNFR2 humanized mice (female). Mice were grouped when tumor volume reached approximately 100 mm^3^. **(A–F)** MC38 tumor bearing TNFR2 humanized mice were treated with 10 mg/kg AN3025 or 10mg/kg mouse PD-1 antibody (mPD-1 Ab) every 3 days intraperitoneally for 7 doses in total. **(A)** Tumor volume measurement during the treatment (n=8 each group). **(B)** Body weight record during the treatment (n=8 each group). **(C)** Representative immunohistochemistry images of CD8 staining on MC38 tumors. **(D)** Quantifications of CD8^+^ T cells per mm^2^ by immunohistochemistry (n=3 each group). **(E)** Representative immunohistochemistry images of CD4 staining on MC38 tumors. **(F)** Quantifications of CD4^+^ T cells per mm^2^ by immunohistochemistry (n=3 each group). **(G, H)** MC38 tumor bearing TNFR2 humanized mice were treated with 3mg/kg AN3025 or 3mg/kg mPD-1 Ab or combination of AN3025 and mPD-1 Ab every 3 days intraperitoneally for 7 doses in total. **(G)** Tumor volume measurement during the treatment (n=8 each group). **(H)** Body weight record during the treatment (n=8 each group). Values were expressed as Mean ± SEM. *P < 0.05; **P < 0.01; ***P < 0.001.

We also compared the anti-tumor effect of AN3025 with anti-PD-1 antibody. We found the anti-tumor effect of AN3025 at 10mg/kg is comparable to that of anti-mouse PD-1 (mPD-1) antibody (10mg/kg) in the MC38 tumor bearing B-hTNFR2 mice in the same study (**Figure 4A**). No body weight change was observed with the indicated treatments (**Figure 4B**). Tumor infiltrating CD8^+^ T cells and CD4^+^ T cells in MC38 tumors from B-hTNFR2 mice were detected with by immunohistochemistry (IHC). AN3025 significantly increased tumor infiltrating CD8^+^ T cells (**Figures 4C, D**) and CD4^+^ T cells (**Figures 4E, F**), while anti-mouse PD-1 antibody showed less effects on tumor infiltrating CD8^+^ and CD4^+^ T cell in the same study (**Figures 4C–F**). This observation might imply different underlying anti-tumor mechanisms for AN3025 and anti-PD-1 antibody, suggesting a combination treatment of AN3025 and anti-PD-1 antibody might achieve better anti-tumor efficacy.

To explore the potential therapeutic strategy, we then treated MC38 tumor bearing B-hTNFR2 mice with the combination of AN3025 and anti-mPD-1 antibody. AN3025 in combination with anti-mouse PD-1 antibody demonstrated stronger *in-vivo* anti-tumor activity than either monotherapy (**Figure 4G**). Neither monotherapies nor the combination therapy caused changes of mice body weight compared with the control group (**Figure 4H**).

## Discussion

Tregs have emerged as a major barrier of anti-tumor immune responses in cancer treatment. Clinical findings have shown that the accumulation of Tregs in the tumor sites correlates with reduced survival, elevated relapse risk and poor prognosis in multiple cancer types (11, 34). Furthermore, TNFR2 expressing Tregs accumulation has been observed and related to adverse clinical outcome in lung cancer (35, 36), ovarian cancer (37, 38), acute myelocytic leukemia (38–40) and cervical cancer (41). Targeting Tregs through blockade of TNFα-TNFR2 signaling pathway has become a novel and attractive strategy for cancer treatment (42). In this study, we report AN3025, a humanized antibody against human TNFR2 and cynomolgus TNFR2, can compete with TNFα for the receptor binding to human TNFR2 and antagonize the TNFR2 receptor activation. The capacity of AN3025 to inhibit Tregs and to enhance T effector cell function has been demonstrated both *in-vitro* and *in-vivo*. As a monotherapy, AN3025 significantly inhibited MC38 and B16F10 tumor growth in TNFR2 humanized murine tumor model. The anti-tumor activity of AN3025 was dependent on the infiltration of CD4^+^ and CD8^+^ T cells, as depletion of CD4^+^ and CD8^+^ T cells abolished the anti-tumor activity of AN3025. In addition, AN3025 in combination with anti-PD-1 antibody demonstrated stronger *in-vivo* anti-tumor activity. Our findings are consistent with previous studies showing blocking TNFR2 with antagonistic antibodies could similarly inhibit Tregs (25, 43) and the surrogate antibody could exert *in-vivo* anti-tumor activity in murine cancer models (44).

AN3025 bound to human TNFR2 and cynomolgus TNFR2 with similar sub nanomolar affinity but didn’t bind to mouse TNFR2 and rat TNFR2. This cross-reactivity feature is not surprising based on sequence analysis of the TNFR2 extracellular domains (ECDs). Protein alignment analysis showed the human TNFR2 ECDs shares only 57.5% and 56% identities with those of mouse and rat TNFR2, respectively. In contrast, human and cynomolgus TNFR2 ECDs are almost identical (a 96.6% identity). The low sequence identity between human TNFR2 and mouse TNFR2 also made humanized murine tumor models an important tool for *in-vivo* efficacy studies of drugs targeting human TNFR2. AN3025 competed with human TNFα to block the TNFα-TNFR2 signaling pathway (**Figure 2F**). Besides, AN3025 is an IgG_1_ format antibody and an intact ADCC activity is critical for AN3025 to exert its maximal *in-vivo* efficacy (data not shown). Therefore, both of blocking and depleting activities are presumably involved in AN3025’s mechanism of action *in vivo*.

Anti-PD-1 antibody monotherapy slightly increased CD4^+^ T cells and CD8^+^ T cells infiltration in our MC38 tumor model, which is consistent with a previous report (45). In contrast, AN3025 treatment increased CD4^+^ T cells and CD8^+^ T cells infiltration to a greater extend, suggesting another potent anti-tumor mechanism. It is known that Tregs can suppress CD4^+^ T cells and CD8^+^ T cells (46, 47). Importantly, both CD4^+^ T cells and CD8^+^ T cells contribute to the anti-tumor immune response (48, 49). To investigate whether the tumor growth inhibition by AN3025 is dependent on T effector cells, we performed a complete depletion of both T cells and found AN3025’s anti-tumor activity is greatly dependent on the T effector cells (**Figure 3D**).

PD-1 blockade has been reported to amplify PD-1^+^ Tregs and induce hyper progressive disease in gastric cancer patients (50). Anti-PD-1 therapy elevated Tregs/Th ratio and promoted squamous cell carcinomas (51). Several murine studies have shown depletion of Tregs with anti-CD25 antibody potentiates anti-tumor activity of anti-PD-1 treatment (52, 53). Targeting Tregs with α-TGFβ antibody or glucocorticoid-induced TNFR-related receptor (GITR) are effective in alleviating the Tregs mediated immunosuppression induced by PD-1 blockade (51, 54). Given the different and complementary mechanisms behind the immune checkpoint inhibitors and Tregs suppression, combination of AN3025 and anti-PD-(L)1 might offer a superior anti-tumor approach. Indeed, in our study, the combination of AN3025 and anti-PD-1 did show improved anti-tumor efficacy over either monotherapy. It would be of interest to further investigate whether AN3025 is able to alleviate the resistance to anti-PD-1 treatments in animal models.

In spite of the preferentially high expression of TNFR2 on Tregs, the immune modulatory role of TNFR2 is multifaceted considering TNFR2 is also expressed on other immune cells including myeloid-derived suppressor cells (MDSCs), mesenchymal stem cells (MSCs), CD8^+^ Tregs, Breg cells, NK cells and CD8^+^T effector cells (2, 55, 56). Similarly, the TNFα-TNFR2 axis promotes proliferation and immunosuppressive function of MDSCs (57), MSCs (58), and Breg cells (59). Thus, the relief of immunosuppression mediated by these cells may also contribute to the *in-vivo* anti-tumor efficacy of AN3025. In addition, TNFR2 is required for the activation-induced cell death (AICD) of activated CD8^+^ T effector cells (60, 61). Therefore, it is hard to exclude the possibility that AN3025, a TNFR2 antagonist antibody, might directly maintain the viability during T cell activation.

In summary, AN3025 is an anti-human TNFR2 antagonistic antibody that is capable of inhibiting Tregs and thus activating anti-tumor response *in-vitro* and *in-vivo*. The potent anti-tumor efficacy of AN3025, either as a monotherapy or in combination with anti-PD-1 antibody, supports its further clinical development for the treatment of various human tumors. Since the TNFR2 expression by immunosuppressive cells exerts protective functions in autoimmunity, the potential risk to trigger autoimmune inflammatory responses by TNFR2 antagonistic antibody should be considered. In our studies, neither body weight nor systemic cytokine release was observed in mice and cynomolgus after AN3025 administration (data not shown), suggesting a relatively safe profile for our TNFR2 antagonist antibody. But for sure the immune-related adverse events will be monitored carefully in the future development of the drug.

## Data Availability Statement

The raw data supporting the conclusions of this article will be made available by the authors, without undue reservation.

## Ethics Statement

The studies involving human participants were reviewed and approved by New York Blood Center. Written informed consent for participation was not required for this study in accordance with the national legislation and the institutional requirements. The animal study was reviewed and approved by the Institutional Animal Care and Use Committee (IACUC) of Beijing Biocytogen Co., Ltd.

## Author Contributions

YC and NH designed research and critically evaluated results. YC, MJ, SW, and SX performed experiments. YC, MJ and SX analyzed data. YC and NH wrote the manuscript. YC, NH, MJ and SX appraised the manuscript. All authors contributed to the article and approved the submitted version.

## Funding

This study received funding from Adlai Nortye Ltd.

## Conflict of Interest

Authors YC, MJ, SW, SX and NH were employed by Adlai Nortye USA Inc.

This study received funding from Adlai Nortye Ltd. The funder had the following involvement with the study: study design, collection, analysis, interpretation of data, the writing of this article and the decision to submit it for publication.

## Publisher’s Note

All claims expressed in this article are solely those of the authors and do not necessarily represent those of their affiliated organizations, or those of the publisher, the editors and the reviewers. Any product that may be evaluated in this article, or claim that may be made by its manufacturer, is not guaranteed or endorsed by the publisher.
