# Supplementary material for: Antagonistic Antibody Targeting TNFR2 Inhibits Regulatory T Cell Function to Promote Anti-Tumor Activity
Source: Front Immunol. 2022 Feb 16;13:835690. doi: 10.3389/fimmu.2022.835690 (PMC8889907; doi:10.3389/fimmu.2022.835690)
Supplement: Supplementary file 1 [file DataSheet_1.docx]

***Supplementary Material***

**Antagonistic antibody targeting TNFR2 inhibits Regulatory T cell function to promote anti-tumor activity**

**Yonglin Chen, Manxue Jia, Sharon Wang, Sherry Xu, Nanhai He***

***Corresponding Author: Nanhai He**

nathan.he@adlainortye.com

**Supplementary Figures**

**
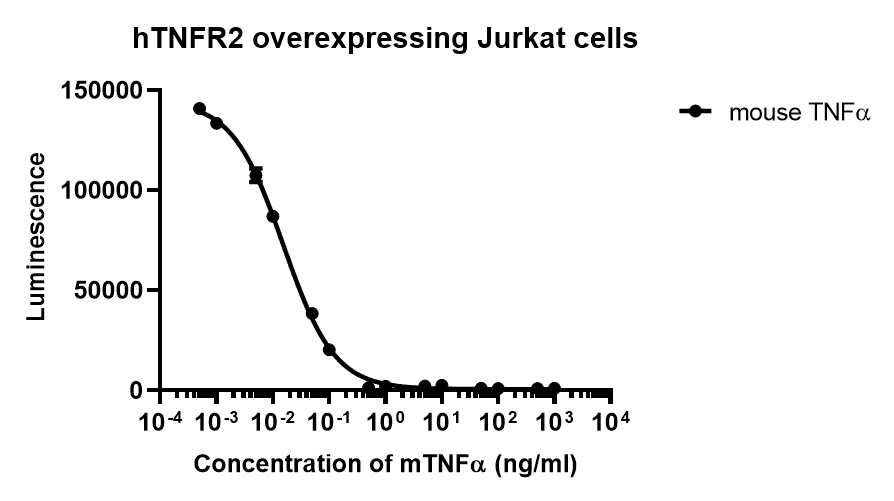
**

**Supplementary Figure 1. Mouse TNFα (mTNFα) induced cell death of hTNFR2 overexpressing Jurkat cells.** Gradient concentration of mouse TNFα (Invitrogen, RMTNFAI) was added to hTNFR2 overexpressing Jurkat cells. The cell viability after 24 hours culture was measured with Promega CellTiter-Glo® Luminescent Cell Viability Assay (Promega, G7570) according to the manufacturer’s instructions. Values were expressed as Mean ± SEM.


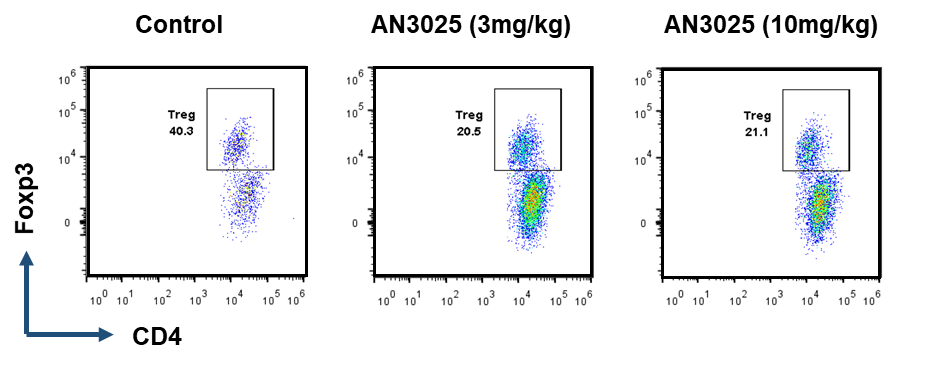


**Supplementary Figure 2. Representative images showing the Treg percentage in CD4^+^ T cells in the MC38 tumors of TNFR2 humanized mice after control or AN3025 administration.** Murine colon cancer MC38 cells (5E5) were implanted subcutaneously into homozygous TNFR2 humanized mice (female). MC38 tumor bearing TNFR2 humanized mice were treated with AN3025 at the dosage of 10mg/kg, 3mg/kg every 3 days intraperitoneally for 3 doses in total. Tregs (CD45^+^CD3^+^CD4^+^ Foxp3^+^) frequency in total CD4+ T cells in the MC38 tumors was quantified by flow cytometry (n=6 each group).


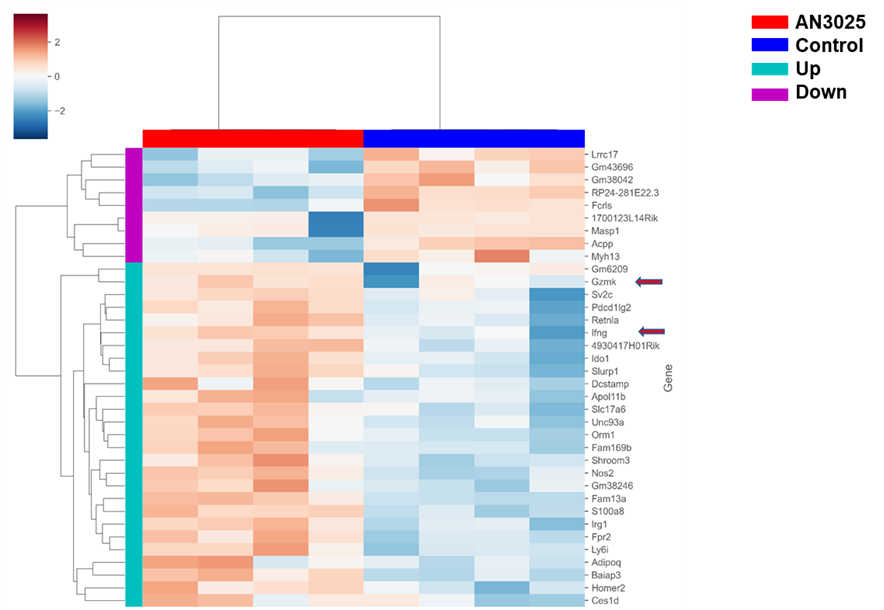


**Supplementary Figure 3. AN3025 increases expression of immune activation genes in the MC38 tumor tissue.** Murine colon cancer MC38 cells (5E5) were implanted subcutaneously into homozygous TNFR2 humanized mice (female). Mice were grouped when tumor volume reached approximately 100 mm^3^. MC38 tumor bearing TNFR2 humanized mice were treated with 10 mg/kg AN3025 every 3 days intraperitoneally for 7 doses in total. MC38 tumor tissues were collected for RNA seq analysis (n=4 each group). Immune activation genes such as Gzmk (gene for Granzyme K) and Ifng (gene for IFN-γ) were upregulated by AN3025 treatment.


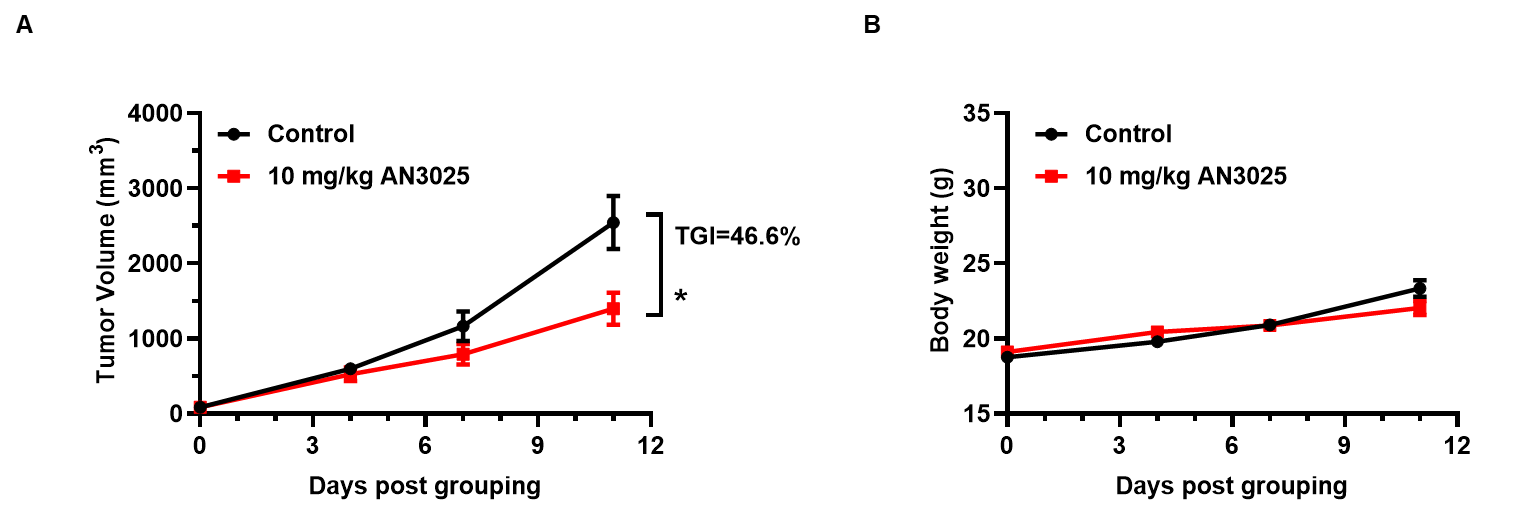


**Supplementary Figure 4. AN3025 significantly inhibits B16F10 tumor growth as a monotherapy in hTNFR2 mouse model.** Murine melanoma cell B16F10 cells were implanted subcutaneously into homozygous humanized TNFR2 mice (female, n=8 each group). Mice were grouped when tumor volume reached approximately 80 mm^3^. Then they were treated with control or 10 mg/kg AN3025 twice per week intraperitoneally for 4 doses in total. (A) Tumor volume measurement during the treatment (n=8 each group). (B) Body weight record during the treatments (n=8 each group). Values were expressed as Mean ± SEM. Statistical analysis of the tumor volumes on the final day of experiments was performed via t-test. *P<0.05.

**Supplementary Table 1 P values of two-way ANOVA analysis of tumor growth in TNFR2 humanized mouse cancer models（*P<0.05; ** P<0.01; ***P<0.001）.**

|  | Groups | P value of Two-way ANOVA analysis |
| --- | --- | --- |
| Figure 3A | 10mg/kg AN3025 v.s. Control | **P=0.007 |
|  | 3mg/kg AN3025 v.s. Control | **P=0.0042 |
| Figure 3D | 10mg/kg AN3025 v.s. Control | **P=0.007 |
|  | 10mg/kg AN3025 v.s. (10mg/kg AN3025+anti-CD4+ anti-CD8) | ***P=0.0001 |
| Figure 4A | 10mg/kg AN3025 v.s. Control | ***P<0.0001 |
| Figure 4G | 3mg/kg AN3025 v.s. Control | ***P<0.0001 |
|  | (3mg/kg AN3025+3mg/kg mPD1 Ab) v.s. Control | ***P<0.0001 |
|  | (3mg/kg AN3025+3mg/kg mPD1 Ab) v.s 3mg/kg mPD1 Ab | *P=0.019 |
|  | (3mg/kg AN3025+3mg/kg mPD1 Ab) v.s 3mg/kg AN3025 | **P=0.0079 |
